# Potentials and challenges of chromosomal microarray analysis in prenatal diagnosis

**DOI:** 10.3389/fgene.2022.938183

**Published:** 2022-07-26

**Authors:** Xijing Liu, Shanling Liu, He Wang, Ting Hu

**Affiliations:** ^1^ Department of Medical Genetics, West China Second University Hospital, Sichuan University, Chengdu, China; ^2^ Department of Obstetrics and Gynecology, West China Second University Hospital, Sichuan University, Chengdu, China; ^3^ Key Laboratory of Birth Defects and Related Diseases of Women and Children (Sichuan University), Ministry of Education, Chengdu, China

**Keywords:** chromosomal abnormalities, conventional karyotyping analysis, chromosomal microarray analysis, copy number variants, genome sequencing, prenatal diagnosis

## Abstract

**Introduction:** For decades, conventional karyotyping analysis has been the gold standard for detecting chromosomal abnormalities during prenatal diagnosis. With the development of molecular cytogenetic methods, this situation has dramatically changed. Chromosomal microarray analysis (CMA), a method of genome-wide detection with high resolution, has been recommended as a first-tier test for prenatal diagnosis, especially for fetuses with structural abnormalities.

**Methods:** Based on the primary literature, this review provides an updated summary of the application of CMA for prenatal diagnosis. In addition, this review addresses the challenges that CMA faces with the emergence of genome sequencing techniques, such as copy number variation sequencing, genome-wide cell-free DNA testing, and whole exome sequencing.

**Conclusion:** The CMA platform is still suggested as priority testing methodology in the prenatal setting currently. However, pregnant women may benefit from genome sequencing, which enables the simultaneous detection of copy number variations, regions of homozygosity and single-nucleotide variations, in near future.

## Introduction

During the past decade, chromosomal microarray analysis (CMA) has been gaining popularity in prenatal diagnosis, especially in the detection of chromosomal abnormalities. Chromosomal abnormalities are responsible for more than 300 types of human syndromes, spanning a wide range of genomic imbalances from polyploidies and aneuploidies (abnormalities in chromosome number) to submicroscopic deletions and duplications (losses or gains of a small portion of chromosomes, known as copy number variants [CNVs]) (Peters et al., 2015). Chromosomal abnormalities occur in approximately 1 in 150 live births and appear in approximately 25% of all miscarriages and stillbirths and in 50–60% of first-trimester miscarriages (Nussbaum et al., 2016).

Generally, the incidence of fetal aneuploidies increases with maternal age (Rose et al., 2020). In contrast, CNVs, which can also lead to unfavorable fetal prognosis, are independent of maternal age and occur in approximately 0.4% of pregnancies (Carvalho et al., 2010). Caused by chromosomal rearrangements resulting in the loss or gain of the dosage-sensitive gene(s)/region(s), CNVs can be categorized into recurrent and non-recurrent aberrations. Recurrent rearrangements, commonly interstitial deletions and duplications, are mediated by nonallelic homologous recombination between flanking sequences with DNA sequence homology (Carvalho et al., 2010). These chromosomal rearrangements, mediated by genomic architecture, generate hotspots for recurrent deletions or duplications that contain unique regions of genomic imbalance, shared among individuals (Carvalho et al., 2010). In contrast, non-recurrent aberrations have varied sizes and breakpoints for each individual and are generated by various molecular mechanisms (Rose et al., 2020). CNVs can directly influence phenotypes and cause diseases by altering gene dosage and/or disrupting gene (Redon et al., 2006). Moreover, CNVs can affect gene expression indirectly through position effects: by changing the regulatory landscape and thus altering crosstalk between alleles or by disclosing recessive mutations (Mikhail, 2014). According to the American College of Medical Genetics and Genomics (ACMG) and Clinical Genome Resource (ClinGen) five-tiered system, CNVs can be divided into the following categories: pathogenic (P), likely pathogenic (LP), variants of uncertain significance (VUS), likely benign (LB), or benign (B) (Riggs et al., 2020).

## Trends in chromosomal testing techniques

Conventional karyotyping has historically been the gold standard for detecting genome-wide chromosomal abnormalities during the prenatal period (Steele and Breg, 1966; Vermeesch et al., 2007). Karyotyping can detect numerical chromosomal abnormalities (polyploidies or aneuploidies), relatively large structural abnormalities microscopically visible to a resolution of approximately 5–10 Mb, balanced or unbalanced translocations, and inversions (Vermeesch et al., 2007). Nevertheless, it has several inherent limitations, such as a relatively long turnaround time owing to the cell culture, the requirement of skilled technicians to perform the analysis, and the inability to detect submicroscopic chromosomal aberrations (Steele and Breg, 1966; Vermeesch et al., 2007).

In addition to karyotyping, various molecular cytogenetic methods have been developed to understand genome architecture in recent decades. For example, fluorescence *in situ* hybridization (FISH) bridges the gap between cytogenetic and molecular approaches. FISH can detect clinically significant chromosomal aberrations in cells during metaphase or interphase (Speicher and Carter, 2005). The major advantage of FISH is the rapid turnaround for the visualization of the physical location of target probe in individual cells (Speicher and Carter, 2005). However, the disadvantage of FISH-based tests is that they cannot balance the detection range and resolution. Chromosome paint-based FISH techniques, for instance, allow the rapid assessment of large chromosomal alterations in the entire genome, but the resolution of the method is limited. DNA probe-based FISH tests are targeted and can only screen individual DNA targets rather than the entire genome (Speicher and Carter, 2005).

With the introduction of CMA, genome-wide detection of CNVs has become possible. CMA can not only identify most chromosomal imbalances detected by conventional cytogenetic analysis but also CNVs with high resolution (Dugoff et al., 2016). Two major microarray platforms are employed in prenatal settings: array comparative genomic hybridization (aCGH) and single-nucleotide polymorphism (SNP) arrays (Schwartz, 2011; Dugoff et al., 2016). In aCGH, chromosomes are represented by large numbers of mapped probes spotted onto standard glass slides (Carvalho et al., 2010). These microarray probes span the whole genome, with particularly dense coverage of clinically relevant genes and regions (Dugoff et al., 2016). Following the hybridization of the fetal DNA sample and normal reference genomic DNA to the target sequences on the microarray, the slide is scanned to measure the fluorescence intensities at each target on the array (Dugoff et al., 2016). The relative intensities of the different fluorescence signals are compared using bioinformatic tools. Cases with duplications had a higher hybridization signal, whereas those with deletions had a lower hybridization signal than the reference sample (Carvalho et al., 2010). In SNP arrays, CNVs are measured using probe signal intensities, as used in the aCGH approach (Schwartz, 2011). SNP probes offer additional advantages. For instance, SNP probes allow for the detection of copy number neutral chromosome abnormalities, such as long stretches of homozygosity that occur owing to uniparental disomy (UPD), consanguinity and maternal cell contamination (MCC) (Schwartz, 2011).

## Microarray application in prenatal diagnosis

CMA is recommended as the first-tier test in the postnatal evaluation of individuals with intellectual disability, developmental delay, autism spectrum disorder, and/or multiple congenital anomalies (Miller et al., 2010). In the prenatal setting, with multiple advantages over conventional karyotyping, CMA is the first-tier recommendation for a prenatal evaluation of fetuses with structural anomalies (American College of Obstetricians and Gynecologists Committee on Genetics, 2013; Dugoff et al., 2016). CMA reliably detects CNVs as small as 50–100 kb in size, which provides better resolution than cytogenetic analysis (Peters et al., 2015). In addition, CMA can be performed on direct fetal samples (uncultured cells), including those obtained from chorionic villus sampling, amniocentesis, or fetal blood sampling, which may lead to a shorter turnaround time (usually within 3–5 days) than karyotyping.

### Diagnostic yield of CMA

Several large-scale studies have compared the diagnostic yield of karyotyping with that of genome-wide CMA for prenatal diagnosis and have shown that a significant proportion of clinically relevant chromosomal aberrations are missed by karyotyping alone (Wapner et al., 2012; Hay et al., 2018; Srebniak et al., 2018). Wapner et al. (2012) reported a prospective study of 4,282 fetal samples for prenatal diagnosis and concluded that chromosomal microarray analysis identified all aneuploidies and unbalanced rearrangements detected by karyotyping. In samples with a normal karyotype, CMA revealed clinically relevant deletions or duplications in 6.0% of patients with a structural anomaly and 1.7% of those with advanced maternal age or positive screening results (Wapner et al., 2012). In a study by Hay et al. (2018) which included 1,475 fetuses with at least one structural anomaly, chromosomal aberrations were detected in 257 pregnancies (17%), of which 12% were karyotype-detectable, 0.7% were possibly partially detectable, and the remaining 4.7% could not be detected by karyotyping (Hay et al., 2018). When assessing the relationship between ultrasonographic soft markers and chromosomal aberrations, it was demonstrated that the overall prevalence of chromosomal aberrations in fetuses with soft markers was 4.3% (107/2,466), comprising 40.2% with numerical chromosomal abnormalities, 48.6% with P CNVs, and 11.2% with LP CNVs (Hu et al., 2021). Various ultrasound results of the fetus, such as ventriculomegaly, short femur, and thickened nuchal translucency, have been evaluated separately, and the advantages of CMA in prenatal diagnosis have been established (Zhang et al., 2019; Wang J et al., 2020; Li et al., 2021).

CMA also has the advantage of detecting chromosomal aberrations in general pregnancy. A meta-analysis assessed CMA in 10,614 fetuses from 10 large studies, reporting pathogenic, clinically significant CNVs in 0.84% (1:119) of cases referred for advanced maternal age or parental anxiety (Srebniak et al., 2018). In 0.34% of normal karyotype fetuses, submicroscopic CNVs associated with developmental delay/intellectual disability were detected; those CNVs may be missed by prenatal ultrasound (Srebniak et al., 2018).

### Limitations of CMA

These results demonstrate that the diagnostic yield of CMA is much higher than that of karyotyping. It has been debated for years whether CMA should be recommended as a first-tier prenatal diagnosis approach to replace the previous recommendations (i.e., either CMA or karyotyping in pregnant women with no positive ultrasound findings). It is worth noting that, currently, karyotyping cannot be completely replaced. Firstly, as CMA cannot detect translocations and inversions, karyotyping should be performed in some situations. For example, CMA can detect trisomy 21 in a prenatal sample, but cannot identify its origin from a non-disjunction event or an unbalanced Robertsonian translocation. In cases of aneuploidies involving Group D/G chromosomes, karyotyping of these fetuses and their parents is essential to determine reproductive risks for future offspring. Secondly, VUS are uncommon genetic alterations with relatively little clinical evidence to evaluate potential pathogenicity effectively. However, their detection by CMA may cause stress and anxiety for the parents, who may need to consider terminating the pregnancy (Levy and Wapner, 2018). Thirdly, CMA is difficult to detect low-level mosaicism due to unbalanced rearrangements and aneuploidy. The copy number, DNA quality, data quality, and size of imbalance, as well as analytical methods, all influence CMA’s sensitivity to detecting mosaicism. The mechanism underlying some genetic imbalances may necessitate the use of karyotyping or FISH. In addition, the fact that karyotyping is much more affordable compared to CMA should be taken into consideration in clinical practice. However, there is a lack of evidence to weigh the potential benefit of higher CMA yields in general pregnancy versus the extra cost of CMA. Lastly, the aCGH cannot identify MCC. In clinical application, quantitative fluorescent polymerase chain reaction (QF-PCR) with short tandem repeat (STR) markers is always performed.

## Crisis and challenge on CMA

Currently, genome sequencing (GS) challenges the status of CMA in prenatal settings; for example, copy number variation sequencing (CNV-seq), genome-wide cell-free DNA (cfDNA), and whole exome sequencing (WES) have been developed in prenatal setting (Table 1).

**TABLE 1:** Summary of prenatal diagnosis/screening methods.

Method	What is detected	Advantage	Disadvantage	Prenatal application
Karyotyping	• Numerical chromosomal abnormalities (polyploidies or aneuploidies)	• Detect chromosomal structure abnormalities	• Relatively long turnaround time	• General population with no positive ultrasound findings
	• Chromosomal abnormalities above 5–10 Mb	• Spend less	• Undetectable submicroscopic chromosomal aberrations	—
CMA	• Numerical chromosomal abnormalities (polyploidies or aneuploidies)	• Detect chromosomal abnormalities not detectable by karyotyping	• Inability to detect molecularly balanced chromosomal rearrangements	• First-tier test when fetal structural anomalies detected
	• CNVs	• Better define and characterize abnormalities identified by karyotyping	• Limitations in the detection of low-level mosaicism	• Fetal with a high risk of UPD
	• ROH	—	• Relatively Expensive	—
CNV-seq	• Numerical chromosomal abnormalities (aneuploidies)	• Detect chromosomal abnormalities not detectable by karyotyping	• Inability to detect molecularly balanced chromosomal rearrangements	• General population with no positive ultrasound findings
	• CNVs	• Relatively cheap	• Inability to detect polyploidies and ROHs	—
			• Less stability, reproducibility, and accuracy	—
NIPT	• Assess the risk of aneuploidies and CNVs	• Non-invasive	• Screening not a diagnostic method	• Screening in the general population
WES	• Exons and flanking sequence of target genes	• All sequence-able exons analyzed	• Only coding sequences, not all genes are equally captured	• Second-tier test when fetal structural anomalies detected
			• Inability to detect CNVs beyond the WES target regions, within poorly covered regions, associated with intragenic regions, or involving single-exon changes	• Situations when a single gene disorder is highly suspected
			• Expensive	—

Abbreviations: CMA, chromosomal microarray analysis; CNVs, Copy number variants; ROHs, Regions of homozygosity; CNV-seq, Copy number variation sequencing; NIPT, Non-invasive prenatal testing; WES, whole exome sequencing.

### Copy number variation sequencing

CNV-seq is uniform, low-coverage genome sequencing based on next-generation sequencing (NGS) at a relatively low price. It seems to be a useful tool for assessing CNVs and has recently been suggested for application in prenatal diagnosis (Dong et al., 2021; Zhang et al., 2021). Zhang et al. (2021), reported that a combination of karyotyping and CNV-seq with an average sequencing depth of 0.08-fold detected an extra 63 cases (0.7%) of pathogenic CNVs in 8,705 cases in populations with a normal karyotype, polymorphism, mutual translocation, or marker chromosome (Zhang et al., 2021). Because of the detection limitations of CNV-seq, two cases of triploids were neglected. The study concluded that the combination of karyotyping and CNV-seq significantly improves the detection rate of fetal pathogenic CNVs (Zhang et al., 2021). Wang H et al. (2020) conducted a consecutive, prospective study to evaluate the yields of CNV-seq compared to CMA. A total of 1,023 women were recruited and CNV-seq identified 124 numerical disorders. CMA detected P/LP CNVs in 121 cases (11.8%) and 17 additional and clinically relevant P/LP CNVs in 17 cases (1.7%). Meanwhile, four cases with regions of homozygosity (ROHs) were missed by CNV-seq. QF-PCR with STR markers was used to exclude MCC and determine polyploidy. The study employed two CMA platforms, namely the 44 K Fetal DNA Chip v1.0 aCGH-based test and an updated 8 × 60 K Fetal DNA Chip v2.0 including SNP probes. The read depth used for CNV-seq was 0.25-fold (Wang H et al., 2020).

The idea that CNV-seq is equivalent or superior to routine CMA has recently been proposed. However, the current situation is insufficient. One of the major considerations for validating an NGS-based test is the read length, the average coverage and depths needed across the genome remain unclear. Short NGS read lengths prevent the detection of variations in repetitive regions with comparable sensitivities (Treangen and Salzberg, 2011). Repetitive DNA sequences are abundantly present in the human genome. Although some repeats appear non-functional, others may play a critical role in human physical development (Treangen and Salzberg, 2011). When repeats are longer than the length of a read, methods must rely on the depth of coverage or paired-end data to determine whether the repeat region is a variant (Treangen and Salzberg, 2011). For instance, suppose that a genome of interest is sequenced to an average depth of 30-fold coverage but a particular tandem repeat with two copies in the reference genome has a 60-fold coverage (Treangen and Salzberg, 2011), under such circumstances, some algorithms incorporating both read-depth and paired-end data for accurate CNVs detection have been employed to improve the estimation of the true copy number of each repeat (Hormozdiari et al., 2009; He et al., 2011; Treangen and Salzberg, 2011). In CNV-seq, relatively shallow average coverage makes it difficult to identify repetitive regions related to specific diseases. For example, the Leri-Weill dyschondrosteosis (LWD)-SHOX deletion is a pseudoautosomal dominant disorder characterized by short stature, mesomelic limb shortening, and a characteristic “Madelung” deformity of the forearms. There are two types of genetic bases: one encompasses the *SHOX* gene deletion, whereas the others are centromeric to *SHOX* within the pseudoautosomal region, and the latter is undetectable by CNV-seq. In addition, although several studies have demonstrated that CNV-seq has a much higher diagnostic yield than CMA, the results should be interpreted cautiously for two reasons: one is that the CMA platforms are varied and have low resolution across different studies; another is the reproducibility of CNV-seq is poor, due to the short read lengths and low sequencing depth. A large, blinded comparative study should be conducted to verify the detection accuracy and stability of CNV-seq and CMA.

It is of great significance to detect UPD in the prenatal setting, as the prevalence of UPD associated with a clinical presentation due to imprinting disorders or recessive diseases ranges from 1 in 3500 to 1 in 5000 (Robinson, 2000). A recent study showed that UPD for all chromosomes occurs with an overall prevalence of 1 in 2000 births, but this can be as high as 1 in 176 among individuals with developmental delay (King et al., 2014; Nakka et al., 2019). UPD cases can be ascertained by testing for copy number abnormalities using CMA platforms with SNP probes. However, assessing ROH can be difficult using low-pass genome sequencing methods. Recently, Dong et al. (2021) demonstrated that all ROHs ascertained by CMA were revealed by low-coverage genome sequencing (4-fold) in 17 clinical samples. In another part of the study, among 1,639 samples (data available from the 1000 Genomes Project), genome sequencing not only consistently detected ROHs but also reported 60 terminal ROHs in 44 cases, including four mosaic ROHs at a level ranging from 50 to 75% (Dong et al., 2021). The authors suggested that for fetuses with a suspected genetic etiology of imprinting disorders or consanguineous mating, low-coverage genome sequencing (4-fold) with ROH detection would be applicable (Dong et al., 2021). However, this view is difficult in the clinical setting. Although the sample size from the 1000 Genomes Project satisfies a comparison study, the heterogeneity (different genome sequencing coverage and CMA density in the two parts of the study) and a very small clinical sample size affected the reliability of the study. Additionally, the coverage and depth of the current CNV-seq are not sufficiently close to satisfying the demand for and rising costs associated with increased sequencing depth. Notably, UPD for specific chromosomes associated with imprinting results in abnormal phenotypes either present or absent in fetal ultrasound (Del Gaudio et al., 2020). Currently, it is better to use CMA (SNP probes contained) rather than other methods for fetuses with structural anomalies, such as growth restriction, overgrowth, and large omphalocele, and for women with positive non-invasive prenatal testing associated with imprinting chromosomes.

### Genome-wide cfDNA testing

Non-invasive prenatal testing (NIPT) has been shown to be a highly sensitive screening test for major fetal trisomies in the last decade. Recently, expanding NIPT covering the entire genome, called genome-wide cfDNA testing, has been applied to detect CNVs beyond major trisomies.

A meta-analysis conducted by Familiari *et al.* demonstrated that the positive predictive value (PPV) was 44.1% in detecting microdeletion and microduplication syndromes by expanding NIPT. However, the small number of cases in each study, the lack of standardized diagnostic confirmation, and different DNA sequencing methods make the results unclear (Familiari et al., 2021). Very recently, a study conducted by Rafalko *et al.* indicated that genome-wide cfDNA testing can provide patients with more clinically relevant information with a PPV of approximately 74.2% (95% CI: 68.1–79.5%) and 71.8% (95% CI: 65.5–77.4%) for “fetal-only” events (Rafalko et al., 2021). However, the opinion on genome-wide cfDNA testing in prenatal setting is controversies. Firstly, it is important to understand that the PPV of NIPT varies depending on the patient’s prior risk of a chromosomal abnormality. Other-than-common benign CNVs are found in approximately 6.7% of fetuses with isolated ultrasound-detected abnormalities (Donnelly et al., 2014). The frequency of other-than-common benign CNVs increased to 13.6% when multiple organ system ultrasound anomalies were observed (Donnelly et al., 2014). All the above studies failed to distinguish the presence or absence of ultrasound anomalies among the true positive population. Stephanie Guseh et al (2021). Assessed the concordance of genome-wide screening and diagnostic testing, indicating that the major limitation of genome-wide screening compared with diagnostic testing is in the population with abnormal ultrasound with *κ* = 0.38 (95% CI, 0.08–0.67), including 5 concordant positives, 4 false positives, 7 false negatives, and 48 concordant negative results, indicating a high residual risk in a false negative population. For patients with a fetal anatomic abnormality, CMA on fetal samples is optimally recommended, whereas NIPT is a choice for low-risk populations. Second, all authorities recommend that a positive NIPT result should be confirmed by an invasive procedure. Although genome-wide cfDNA screening testing has a PPV of approximately 74.2%, the population that requires invasive procedures increases with the emergence of false-positive results. The initial purpose of NIPT was to decrease the need for invasive testing; however, the current situation is contrary to that intention.

As P/LP CNVs occur in approximately 1.7% of patients with a normal ultrasound examination (Wapner et al., 2012), the concordance between genome-wide screening and diagnostic testing in a population without abnormal ultrasound findings is worthwhile. Further is required to comprehensively estimate the clinical implementation of genome-wide cfDNA testing.

### Whole exome sequencing

WES is a technology used to interrogate the genome at the nucleotide level to identify variants in a single-gene disorder, and is empirically proposed to be more informative than CMA. WES sequences exons and flanking intron sequences with high coverage. Although WES provides more genomic information, the current best practices for CNV detection still require CMA. Studies have been conducted to assess the performance of WES data in CNV detection and have identified several potential blind spots: CNVs beyond the WES target regions, or within poorly covered regions, as well as CNVs associated with intragenic regions, or involving single-exon changes (Retterer et al., 2015; Royer-Bertrand et al., 2021).

Emerging evidence supports the benefits of WES when fetal structural anomalies are detected. Mellis et al. (2022) conducted a meta-analysis to determine the diagnostic yield of WES for prenatal diagnosis of fetal structural anomalies. The study summarized that the pooled incremental yield of WES over CMA/karyotype from all studies was 31% (95% CI 26-36%, *p* < 0.0001). An updated statement released by the International Society for Prenatal Diagnosis recommends using genome-wide sequencing for prenatal diagnosis, which suggested that the use of WES should follow indications in prenatal clinical diagnosis. For those with no genetic diagnosis found after CMA, pregnancy with a fetus having a single major anomaly or multiple organ system anomalies will benefit from WES or other genome sequencing methods (Van den Veyver et al., 2022). There is currently no evidence supporting WES as a routine test for indications other than fetal anomalies or a single gene disorder being highly suspected. It should also be noted that the interpretation of WES results is challenging in prenatal diagnosis settings. For instance, reporting uncertain results hinders clinical interpretation, and there is no universal consensus on the management of incidental and secondary findings. Currently, CMA is still the first step for a fetus with structural anomalies, and WES could be a further step when normal chromosomes are detected after detailed genetic counseling (Monaghan et al., 2020; Van den Veyver et al., 2022).

## Conclusion

CMA is recommended when fetal structural anomalies are detected. Whether this is a first-tier recommendation in general pregnancy is disputable. Genome sequencing, which provides the ultimate genetic test for the detection of more informative genomic variation in a single assay, challenges the status of CMA in the prenatal setting, but until now, in a practical context, it has been insufficient to replace CMA. CMA platforms with SNP probes are currently superior to CNV-seq in detecting chromosomal abnormalities, such as the combined detection of CNVs, MCC, and ROHs, which are common and significant in prenatal settings. Moreover, the stability, reproducibility, and accuracy of CNV-seq remain unclear. The prenatal use of CNV-seq is at risk, especially in cases with positive ultrasound findings or positive noninvasive prenatal testing associated with imprinting chromosomes. Genome-wide cfDNA testing with non-invasive features has attracted the attention of researchers for assessing the utility of CNV detection. However, the application of genome-wide cfDNA testing in the prenatal setting is controversial because of its low concordance with diagnostic results, especially in fetuses with structural anomalies. WES with a scope at the nucleotide level is recommended as a second-line test when fetal structural anomalies are detected, as some CNVs may be missed by the method.

In conclusion, the development of molecular tests has changed prenatal diagnosis, and the CMA platform with SNP probes has been suggested in the prenatal setting. However, the trend toward GS, which is used to identify CNVs along with SNVs and ROHs simultaneously in a single assay, is unstoppable and will be of great benefit to pregnant women in the future by providing more useful information with rapid turnaround times and at acceptable prices.
